# No Differential Effects of Neural and Psychological Explanations of Psychopathy on Moral Behavior

**DOI:** 10.3389/fpsyg.2018.01317

**Published:** 2018-07-31

**Authors:** Robert Blakey, Adrian Dahl Askelund, Matilde Boccanera, Johanna Immonen, Nejc Plohl, Cassandra Popham, Clarissa Sorger, Julia Stuhlreyer

**Affiliations:** ^1^Centre for Criminology, University of Oxford, Oxford, United Kingdom; ^2^Department of Psychiatry, University of Cambridge, Cambridge, United Kingdom; ^3^Department of Psychology, King’s College London, London, United Kingdom; ^4^Faculty of Medicine, Imperial College London, London, United Kingdom; ^5^Department of Psychology, University of Maribor, Maribor, Slovenia; ^6^Department of Experimental Psychology, University of Oxford, Oxford, United Kingdom; ^7^Division of Psychology and Language Sciences, University College London, London, United Kingdom; ^8^Center for Anesthesiology and Intensive Care Medicine, University Medical Center Hamburg-Eppendorf, Hamburg, Germany

**Keywords:** neurocriminology, attitude change, belief in free will, psychopathy, science communication

## Abstract

Research in neurocriminology has explored the link between neural functions and structures and the psychopathic disposition. This online experiment aimed to assess the effect of communicating the neuroscience of psychopathy on the degree to which lay people exhibited attitudes characteristic of psychopathy in particular in terms of moral behavior. If psychopathy is blamed on the brain, people may feel less morally responsible for their own psychopathic tendencies. In the study, participants read false feedback about their own psychopathic traits supposedly inferred from their Facebook likes, described either in neurobiological or cognitive terms. Participants were randomly allocated to read that they either had above-average or below-average psychopathic traits. We found no support for the hypothesis that the neuroscientific explanation of psychopathy influences moral behavior. This casts doubt on the fear that communicating the neuroscience of psychopathy will promote psychopathic attitudes.

## Introduction

With a long history of presenting scientific testimony in the courtroom (Golan, 1999), the future criminal justice system could be informed more broadly by ever-growing experimental science. In particular, the system may be informed by neurocriminology, which aims to identify the neurobiological correlates of criminal behavior (Umbach et al., 2015). If neurocriminology is integrated into the criminal justice system, this transition will take place within the view of offenders. Consequently, offenders will learn more about the cognitive, genetic and neurobiological predictors of their own antisocial behavior and mental health conditions. Hence, with advancements in science and technology, offenders may develop an understanding of the otherwise hidden contributors to their criminal behavior. While qualitative researchers have begun to probe the response of offenders to neurocriminology (Horstkötter et al., 2012, 2014), no study has considered its effects on moral behavior. Hence, the current research measured the behavioral response of lay people to the feedback about one psychopathic trait: moral alarm.

### Moral Alarm

Here, moral alarm is defined as the anxiety experienced in the process of causing harm. Psychopathy is characterized by a lack of moral alarm, or more generally a lack of empathy for the suffering of others (Aniskiewicz, 1979; Hare, 1991). Having a higher moral alarm reduces the probability of people making utilitarian decisions; for example, when faced with the decision to sacrifice one life to save five lives, empathy for the one life typically inhibits the decision to save the five lives (Thomson, 1985; Greene et al., 2001, 2008). In contrast, the utilitarian choice is more likely to be made by people who report feeling less empathy (Bartels and Pizarro, 2011; Conway and Gawronski, 2013; Gleichgerrcht et al., 2013) and people who score higher on the psychopathy scale (Koenigs et al., 2012). While Cima et al. (2010) observed no such effect of psychopathy, this may be attributable to the small sample size and lenient categorization criteria (Koenigs et al., 2012).

### How Could Neuroscience Undermine Moral Behavior?

There are four plausible mechanisms by which neuroscience could undermine moral behavior. First, by providing a causal explanation of behavior, neuroscience may reduce the burden of taking moral responsibility or guilt for the act; hence the expected cost of behaving immorally may be reduced. Second, neuroscience may promote belief in fatalism and therefore reduce the perceived capacity to behave morally even if one aspired to behave morally (Dar-Nimrod and Heine, 2011; Miles, 2013). Third, neuroscience could simply increase the believability of behavioral feedback, thereby strengthening the probability of people conforming their moral behavior to the neuroscientific feedback. While research has considered the effects of communicating neuroscience *as part of* some larger argument, for example, against free will, no empirical study has considered the effects of communicating neuroscience *per se*. Hence, the current study permitted neuroscience to pose effects through any of these four mechanisms.

### Neuroscience as a Challenge to Belief in Free Will

Neuroscience does not distinguish between the brain and the person; instead it adopts the physicalist position that ‘all psychological states are also biological ones’ (Monterosso and Schwartz, 2012). However, lay people appear to be intuitively dualistic, implicitly perceiving the brain to be separate from the mind (Bloom, 2004; Forstmann and Burgmer, 2015). It was therefore hypothesized that the current participants would consider the brain-based explanation intuitively to be different from the mind-based explanation. Specifically, people may consider the ‘brain’ to be a less controllable cause of offending than the ‘mind.’ Hence neuroscientific arguments could challenge specific beliefs that individuals hold, such as belief in free will (Harris, 2012).

There are two possible means by which neuroscience could challenge belief in free will. First, the brain could be considered an unconscious cause of behavior (Nahmias et al., 2007; Shepherd, 2012). Second, even if people do consider the brain to compute conscious intent and desire, the cause of conscious brain activity might appear uncontrollable; for example, a product of genes and life experiences or features of the environment that are ultimately products of chance (Greene and Cohen, 2004; Morse, 2004). Therefore, people may consider the brain to be beyond conscious *or* free choice; either way, neuroscience is capable of challenging belief in free will (Shariff et al., 2014). With reduced belief in free will, people may experience less incentive or obligation to act morally.

### The Behavioral Consequences of Belief in Free Will

No empirical study has tested the effects of communicating neuroscience on moral behaviors such as altruism. However, researchers have observed moral consequences of challenging belief in free will. In this avenue of research, participants are typically asked to read a passage of text that challenges their intuitive belief in free will. As a result of reading this passage, participants are more likely to cheat passively (Vohs and Schooler, 2008; study 1) or proactively (study 2), less likely to help a person in need (Baumeister et al., 2009; studies 1 and 2), more likely to act aggressively (Baumeister et al., 2009; study 3), more likely to conform to the opinion of others (Alquist et al., 2013), and less likely to co-operate for the collective good (Protzko et al., 2016). Therefore, challenges to free will may undermine the pursuit of moral behaviors.

Importantly, deterministic messages reduce co-operation only when participants are required to make their moral choices quickly rather than slowly; and hence intuitively rather than deliberatively (Protzko et al., 2016). This may be explained by the finding that deterministic messages sometimes reduce implicit, yet not explicit, attributions of agency (Lynn et al., 2014). It is important to caveat this research with two studies that have reported null effects of challenging belief in free will on cheating (Open Science Collaboration, 2015) and stealing (Monroe et al., 2017). Nevertheless, there is clearly a need to address *when* challenges to free will *do* promote immoral choices (Baumeister, 2008). This study aims to investigate, in addition to whether neuroscientific explanations reduce free will belief, if this in turn will have consequences for different moral behaviors, such as cheating in a die roll task.

However, neither this study nor prior evidence suggest such effects are mediated by conscious reasoning. When deterministic messages have been found to reduce the probability of people ‘deciding’ to exercise self-control, the affected mechanism for that decision is typically unconscious (Rigoni et al., 2015) and the decision is made in far less time than conscious reasoning would permit; for example, in less than 2 s (Lynn et al., 2013) or even 1.28 s (Rigoni et al., 2012). Although people can perceive the reductions in self-control induced by deterministic beliefs (Rigoni et al., 2012), the philosophy of determinism has also been found to delay *preconscious* motor signals in the brain (Rigoni et al., 2011) and not the conscious equivalent of such signals (Rigoni et al., 2015). Since conscious reasoning is therefore not a necessary cause of the behavioral effects, the current argument becomes more plausible: a deterministic message *could* reduce unconsidered moral motivation to behave honestly.

### Behavioral Consequences of Belief in Fatalism

While no researcher has studied the behavioral consequences of belief in fatalism, extensive empirical research has considered the consequences of believing that personality traits can or cannot be changed (Dweck, 2006). This work is founded upon the distinction between the fixed mindset or entity theory – the belief that traits are fixed entities that cannot be changed – and the growth mindset or incremental theory – the belief that intelligence can grow with the incremental investment of effort (Hong et al., 1999). While the empirical focus has been the implications of perceiving intelligence as fixed or incremental, we predict equivalent effects would be observed from attributions for criminal behavior: the entity theorist would believe the causes of criminal behavior cannot be changed, whereas the incremental theorist would believe that those causes can be changed if only offenders invest effort in changing their beliefs and their environment. In turn, the entity theorist who offends may invest far less effort in opportunities for rehabilitation, since such effort would appear to be futile.

Although neurocriminology constitutes evidence of neither entity theory nor fatalism, it is possible that lay people construe neurocriminology as support for entity theory. The communication of neurocriminology may drive offenders to adopt an entity theory of offending and in this respect, may promote the belief that it would be futile to invest effort in rehabilitation. In the study of academic motivation, entity theorists have less intrinsic motivation to continue an intellectual task after performing poorly on the task (Dweck, 2006). This failure to persist follows from their tendency to interpret their poor performance as a reflection of their inherent traits. Whereas entity theorists make excuses, blame others and lose confidence, incremental theorists interpret failure as the opportunity to improve their future behavior. The present study seeks to extrapolate this theory to attributions for antisocial traits: neurobiological explanations of antisocial traits may develop an entity theory of such traits, thereby reducing efforts to squash those traits.

### The Believability of Neuroscience

Neuroscientific feedback could simply be more believable than cognitive feedback, even when the source of that feedback remains the same. In the neuroscientific context, people may firstly be more convinced of the existence of moral alarm. Secondly, they may be more convinced that *their* moral alarm is as weak or as strong as the feedback states.

Regarding the first point, people may be more likely to believe that a moral alarm exists in the brain (relative to the mind). The addition of circular neuroscience increases the perceived credibility of cognitive science (Weisberg et al., 2008, 2015). The current study extended this research by supplementing the cognitive explanation with neuroscientific facts, ensuring that no reference to brain regions were mentioned, thereby avoiding any effects of jargon.

Second, the direction of the feedback may be more believable. Alquist et al. (2013) observed that participants were more likely to conform to others’ judgments after their belief in free will had been challenged, or their belief in determinism had been increased. Therefore, challenges to free will may substitute self-control with social control, such that decisions become more subject to the group consensus. The current study extended this research by testing whether neuroscience, as a challenge to free will, could induce conformity toward the scientific judgment that their moral alarm is stronger or weaker than average. In contrast, the feedback presented in cognitive terms may be less compelling.

### Facebook Analysis

Given the practical and ethical issues implicated in measuring the response of real offenders to personalized scientific feedback, this study focused on analysing how lay people respond to such feedback. In the current age of technology, social media has generated major new opportunities to analyze behavior online, particularly through the capture of the so-called ‘digital footprints’ left by millions of people on social networks. By using these sources of big data, researchers are generating opportunities for people to receive personalized data-driven feedback concerning their psychological and physical health. For example, Kosinski et al. (2015) analyzed the data of millions of Facebook users to create an algorithm capable of predicting users’ gender, sexuality, age, personal interests, and political views, only based on their Facebook profiles (including statuses, likes, etc.). Such algorithms have also been used to identify the possible psychopathic traits of ordinary people (Garcia and Sikström, 2014).

The method of the current study is largely based on the idea that trait information can be inferred from an individual’s Facebook profile. Specifically, participants will be given false feedback regarding their psychopathic traits after entering their Facebook login details: such traits would described as either below- or above-average. The effect of providing such feedback on their moral behavior will then be measured. If individual scientific feedback is capable of changing the moral behavior of lay people, one might also expect this feedback to influence the moral behavior of offenders who receive such feedback in the future. Hence, the findings of our study will pose implications for the real world, in which personalized neuroscience might 1 day influence how offenders are treated after trial, how offenders explain their own criminal behavior and therefore their own likelihood of reoffending (Maruna and Copes, 2005).

### The Current Study

This online experiment aimed to capture the behavioral consequences of communicating the neuroscience of psychopathy. Participants read that their moral alarm, expressed in neurobiological or cognitive terms, was either 18–22% stronger or 18–22% weaker than average. This study extended previous research in four respects:

(1) Neuroscience was presented on its own, instead of a direct challenge to free will or a direct assertion of determinism.
(2) Neuroscience was presented as an explanation of moral alarm rather than behavior in general.
(3) Participants read about *their own* moral alarm traits rather than behavior in general.
(4) Participants read that this trait feedback had been generated from an analysis of their Facebook likes.

### Hypotheses

It was predicted that participants who read that their moral alarm was weak, this being a psychopathic trait, would be more likely to display psychopathic tendencies in the behavioral tasks. Specifically, three hypotheses were proposed:

H1: Participants who read that their moral alarm was weak (rather than strong) would be more likely to cheat and display utilitarian reasoning.
H2: The behavioral effects of the false feedback (H1) would be greater when the feedback was expressed in neurobiological (rather than cognitive) terms.
H3: The behavioral effects of the false feedback (H1) would be mediated by self-control, belief in dualism, determinism and free will, and the guilt experienced in response to the utilitarian dilemmas.

## Materials and Methods

### Design

This online experiment adopted an independent groups design. Two variables were manipulated between groups: the communicated degree of psychopathic traits (above-average or below-average) and the description of such traits either in neurobiological or cognitive terms. Participants responded through the online survey platform, Qualtrics. The study received ethical approval from the University of Oxford Central Ethics Committee. The study design was also published as a Protocol article in Frontiers in Psychology (Blakey et al., 2017).

### Participants

The sample included 760 participants (68.1% females, 29.8% males, 2.1% other) from 47 countries, with a mean age of 25.6 years (*SD* = 7.5). Since we expected a small effect size (Cohen’s *d* = 0.2), and set the probability of Type I error to 5% and the probability of Type II error to 20%, we initially aimed to recruit approximately 800 participants. In the given time frame, we successfully obtained data from about 95% of respondents intended – a share that was sufficiently close to our initial goal.

Participants were recruited using adverts posted on social media and distributed through the mailing lists of university departments and societies in Austria, Germany, Italy, Slovenia, Finland, Norway, and the United Kingdom. Anyone aged 18 or older and capable of understanding English was eligible to participate; before the study began, participants were asked to rate their English competence. Upon completing the study, participants were entered into a lottery, with the chance to win up to £60.

### Procedure

From the outset, participants were informed that the aim of the study was to test a new method of measuring personality traits; specifically, this involved a computerized analysis of a person’s Facebook likes, able to infer a trait known as moral alarm that psychopaths lack, this trait representing the degree of anxiety that a person experiences while committing immoral acts (Blair, 2006).

The study was split into three sections. In the first section, participants completed the psychopathy scale from the Short Dark Triad (SD3) questionnaire, whereby participants indicated their agreement (1 = *strongly disagree*, 5 = *strongly agree*) with nine statements describing psychopathic traits (Jones and Paulhus, 2014). An internal consistency measure of reliability, run on R with package rho (Lipsanen, 2015), was intermediate (Tarkkonen’s rho = 0.74). We have used Tarkkonen’s rho to measure the internal consistency instead of Cronbach’s alpha, because Cronbach’s alpha’s strict assumptions, such as equal correlations and standard deviations between the items, are rarely fulfilled. This can cause either overestimation or underestimation of reliability (Vehkalahti et al., 2006). Vehkalahti et al. (2006) have shown that Cronbach’s alpha is a special case of Tarkkonen’s rho, and comparisons have indicated that Tarkkonen’s Rho gives more accurate reliability estimates than Cronbach’s alpha (Vehkalahti et al., 2006). A confirmatory factor analysis was ran on R with Lavaan package (Rosseel, 2012) to test the fit of the psychopathy questionnaire data on the theoretical structure presented by Jones and Paulhus (2014). This four factor (“Callous Affect,” “Short-Term Manipulation,” “Antisocial Behavior,” and “Erratic Lifestyle”) model did not indicate good fit, RMSEA = 0.07, TLI = 0.84. Exploratory factor analyses were therefore conducted to examine the structure of psychopathy. The item “I enjoy having sex with people I hardly know” consistently showed low factor loadings to all factors (≤0.30) and was dropped from the scale. This item might have been confounded by cultural differences, making it unfit as an indicator of psychopathy in this sample. A structure of two orthogonal factors with varimax rotation indicated good fit (RMSEA = 0.05, TLI = 0.93) and all the loadings were sufficient (>0.30). A cutoff of 0.90 for Tucker Lewis Index has conventionally been considered to be sufficient to discriminate the unfit structure models, even if more conservative limits have been suggested (Hu and Bentler, 1999). Based on the factor loadings and interpretation, the first factor was termed “manipulativeness and lack of empathy.” This factor consisted of 5 items related to the antisocial aspects of psychopathy, e.g., “I’ll say anything to get what I want.” Another factor, named “impulsivity,” consisted of 2 items describing the impulsive tendencies linked to psychopathy, e.g., “I avoid dangerous situations” (inverse). In addition, the 8th item, “People often say I’m out of control,” had equally high loadings to “manipulativeness and lack of empathy” and “impulsivity.” This supports the notion of two latent factors, as this item has both antisocial and impulsive elements. This 8th item was added into the “impulsivity” score to balance the number of items in each factor. Conceptualizations of psychopathy have identified deficits in affect and self-control as central elements (Hare and Neumann, 2008). Thus factors covering these key elements form a valid coverage on the psychopathic traits. The scores from these two factors are added up to form an overall score on psychopathy. To assure that responses to this questionnaire were not affected by our manipulation or other tasks in the survey, the SD3 was always the first measure to be completed by participants. By including this scale, it became possible to test the effects of the moral decision making manipulation, while controlling for actual psychopathy.

In the second part of the study, participants read about several new concepts. To test their comprehension of these concepts, a single multiple choice question was included at the end of every page of the descriptive text. First, participants read either a cognitive or a neurobiological explanation of moral alarm. The explanations were adapted from the explanations presented by Aspinwall et al. (2012), whose explanations are based on the neurocognitive model of psychopathy (Blair, 2006). In contrast to the original explanations, references to genetics were removed, such that the two explanations (for the neurobiological and cognitive conditions, respectively) were scientifically equivalent, differing by only a few single words.

The explanations read as follows, with the manipulated brain-based and mind-based words shown in italics: ‘The *brain’s/mind’s* moral alarm // Extensive research shows that human *brains/minds* have a moral alarm. The moral alarm is the *physical/psychological* system that produces feelings of anxiety when you behave badly. // When humans behave badly, their *brain/mind* normally generates particular *electrical signals and chemical reactions/thoughts and emotions* that produce feelings of anxiety. The purpose of this anxiety is to *physically/psychologically* reduce your desire to behave badly.’

Subsequently, participants were told of the difficulty in inferring the degree to which a person experiences moral alarm, simply by asking the person directly. People lack insight into their own character and are biased toward presenting themselves in a socially desirable light. In contrast, implicit behaviors, such as liking particular Facebook groups and pages, may provide a more accurate source of information about personality traits, including the experience of moral alarm.

Participants were then required to watched a video, explaining that researchers have designed a computer algorithm able to analyze a person’s Facebook likes to infer even intimate characteristics of the Facebook user (e.g., intelligence, ethnicity, and political views). The video combined a variety of national media coverage of the innovative Facebook research conducted by Kosinski et al. (2013). After watching the video, participants entered a shortened and anonymized web link to their Facebook profile page, supposedly so that their own Facebook likes could be analyzed by the computer algorithm. In order to ensure systematic anonymity, the entered web link was not analyzed nor used in any other manner by the researchers, but was purely used to realistically ensure that participants believed the study process. However, when being debriefed online, participants were given a link to the website where they could access the computerized analysis ^1^. After entering the link to their profile, participants received false feedback about their own moral alarm. Moral alarm was deliberately described without referring to psychopathy in order to avoid triggering the popular negative conception of psychopathy. Participants were randomly allocated to read one of four types of feedback, stating that their own moral alarm was either 18–22% stronger or 18–22% weaker than the average moral alarm and again, either in neurobiological or cognitive terms.

The explanations read as follows, with the four experimental conditions shown in italics: ‘Your *brain/mind* // We would now like to tell you more about people like you, who have an 18–22% *stronger/weaker* moral alarm than the average person. // The moral alarm is the *physical/psychological* system in the *brain/mind* that produces feelings of anxiety when you behave badly. The purpose of this anxiety is to *physically/psychologically* reduce your desire to behave badly. Since your moral alarm is 18–22% *stronger/weaker* than the average moral alarm, you are 18–22% *less/more* likely to behave badly than the average person. This is true of anyone with an 18–22% *stronger/weaker* moral alarm. // People have moral alarms of different strengths because of *physical/psychological* differences in how their *brains/minds* work. When people with a *brain/mind* like yours behave badly, their *brain/mind* generates *more/less* of the *electrical signals and chemical reactions/thoughts and emotions* that produce feelings of anxiety. // Therefore, people with a *brain/mind* like yours feel 18–22% *more/less* anxious when they behave badly. Consequently, people with a *brain/mind* like yours are 18–22% *less/more* likely to behave badly.’

The third section of the study required participants to complete measures for the hypothesized mediators and the outcome variables, the order of which were counterbalanced. In other words, half of the participants responded to the mediators, followed by the outcome variables. The remaining half responded to the outcome variables, followed by the mediators. In respect to the mediators, participants completed the Determinism Subscale and the Free Will Subscale of the Free Will Inventory, and a measure of dualistic beliefs. For the Determinism Subscale of the Free Will Inventory (Nadelhoffer et al., 2014), participants indicated their agreement (1 = *strongly disagree*, 7 = *strongly agree*) with five statements in support of determinism (e.g., ‘Every event that has ever occurred, including human decisions and actions, was completely determined by prior events’). In the current study, this Determinism Subscale exhibited high internal consistency (Tarkkonen’s rho = 0.87). For the Free Will Subscale of the Free Will Inventory (Nadelhoffer et al., 2014), participants also indicated their agreement (1 = *strongly disagree*, 7 = *strongly agree*) with five statements regarding free will (e.g., ‘People ultimately have complete control over their decisions and their actions’). The Free Will Subscale also exhibited high internal consistency (Tarkkonen’s rho = 0.84).

In order to measure dualistic beliefs, participants completed a modified version of the thought experiment designed by Forstmann and Burgmer (2015). The authors asked participants to imagine that scientists had developed a device capable of duplicating any person in a matter of seconds, using highly advanced technology. Participants read that after placing a person in a special chamber, a computer could scan the entire person, every molecule and atom, and store the information digitally. Subsequently, the information could be used to recreate the scanned person from basic chemical elements in a second chamber, resulting in a 100% identical copy of the scanned person, with a 100% success rate.

In contrast to the original task, the participants in this study were asked to imagine that *they* were placed in the chamber and duplicated. Participants then indicated their agreement (1 = *definitely no*, 7 = *definitely yes*) with six statements regarding whether their duplicate shared properties of themselves. Three of the statements concerned psychological properties of relevance to the experimental manipulation (e.g., ‘Is the moral alarm in your duplicate the same strength as the moral alarm in you?’). The remaining three statements concerned physical properties (e.g., ‘Does your duplicate have the same eye color as you?’), which had relatively low internal consistency (Tarkkonen’s rho = 0.73), as the scale for mental properties exhibited high internal consistency (Tarkkonen’s rho = 0.93).

If people do separate the mind from the body, the participants should ascribe the same physical, yet different psychological, properties to the duplicate of their original selves. A confirmatory factor analysis on two orthogonal factors indicated a bad fit, TLI = 0.90, RMSEA = 0.14. An exploratory two factor structure indicated that one of the physical scale items had a stronger factor loading to the same factor as the mental scale items. If this item would have been deleted, Tarkkonen’s rho would have remained low, rho = 0.66. The dualism scale was therefore formed as a subtraction of the three mental items from the three physical items as planned. The consistency of the answers on the physical items was not as good as could have been expected, and this casts caution on the reliability of the formed dualism scale.

Next, participants completed a test of self-control, using a modified online version of the famous marshmallow test (Mischel and Ebbesen, 1972). Although every participant was entered into a lottery for taking part in the study, participants had to decide the timing of that prize, this being whether they would prefer to know the lottery outcome immediately after completing the study, or 3 months later. The incentive for choosing the latter option – the option that required greater self-control – was a £60 raise in the prize money.

To measure utilitarian reasoning, participants responded to three moral dilemma. In all of these dilemmas, participants had to decide whether to initiate one death in order to save a greater number of other people. For the first dilemma, participants were given the crying baby dilemma (Greene et al., 2001), whereby they had to decide whether to smother their hypothetical child to death to avoid catching the attention of enemy soldiers and thereby save several other lives, including their own. For the second dilemma, the standard trolley dilemma (Foot, 1978), was administered to participants, who had to decide whether to allow a runaway trolley to kill five workmen or to pull a lever capable of switching the direction of the trolley, such that only one workman was killed instead. For the last dilemma, participants were given the footbridge dilemma. In the footbridge dilemma (Thomson, 1985), participants decided whether to push a heavy man off a railway bridge to block a runaway trolley from killing five workmen. After making each decision, participants indicated their agreement (1 = *strongly disagree*, 6 = *strongly agree*) and their guilt in making such decisions.

To measure participants’ dishonesty, an online version of the die-under-the-cup task was administered (Shalvi et al., 2011). Participants rolled a virtual die three times, under the instruction that the outcome of their first throw would determine the value of the lottery prize for participating in the study: the higher the outcome, the greater the value of the prize (1 = £10, 2 = £20, 3 = £30, 4 = £40, 5 = £50 and 6 = £60). Hence, participants were given an incentive to misreport the outcome of their first roll to increase the value of the lottery prize. In place of a physical die, participants simply clicked a button and read that a virtual die had been thrown, with a particular outcome obtained. The outcomes of the virtual die were always a two, then a six, and finally a three. Fixing the outcomes allowed the analysis of deception at the individual (and not just the group) level. After rolling the die three times, participants were asked to report the outcome of their first roll (from 1 to 6) within a time frame of 30 s. Participants were warned that if they failed to report the outcome of the roll within 30 s, the prize would be fixed to the minimum of £10. The 30-s time limit was visible from a ticking counter and was included because of evidence that time pressure increases dishonesty. It was important to design the task such that potential floor effects (i.e., near zero rates of dishonesty) were avoided (Shalvi et al., 2012).

The order of presenting each mediator and each outcome variable was randomly determined for every participant. This further instance of counterbalancing was important given the potential for order effects: participants who read that they had a strong moral alarm may have subsequently made more moral decisions, yet that very display of moral decision making may have reduced their subsequent motivation to behave morally. Following moral behavior, people *become* more likely to behave immorally (Blanken et al., 2015). This effect of moral licensing may result from a change in the capacity or the motivation to act morally (Merritt et al., 2010). The random order of task presentation was designed to overcome such effects.

Before concluding the study, participants indicated the believability (1 = *strongly disbelieved*, 6 = *strongly believed*) of the (below-average or above-average) feedback about their moral alarm and the (neurobiological or cognitive) explanation of their moral alarm. Participants also provided basic demographic information: age, gender, nationality, field of study and which device they used to complete the study. Participants could also enter their email address to receive the lottery prize should they win. The email address was stored separately to their survey responses to protect the anonymity of the data. Finally, participants read an extensive debrief about the false feedback.

## Results

Thirty-five percentage of the sample (*N* = 266) reported disbelief or strong disbelief to one or both manipulations, but these participants were kept in the analyses to avoid any bias that could have arose from unequal distribution of disbelievers and their background variables between the experimental conditions. To first establish any significant effects, between subjects MANOVAs and MANCOVAs were run. Only partial mediation was assumed, and therefore the direct effects were investigated. It has been argued that even when using an omnibus test such as ANOVA, the *p*-value should be corrected for multiple comparisons (Cramer et al., 2016). Therefore, the significance level was set with Bonferroni correction to *p* = 0.0023, correcting for the 21 *post hoc* analyses conducted. The dependent variables were analyzed using a between-subjects MANCOVA first. The Strength of the moral alarm [Weak, Strong] and the Level of Explanation [Cognitive, Neurobiological] were entered as between-subjects factors. The actual degree of psychopathy and the believability of both manipulations were entered as control variables. There were seven outcome variables: belief in free will, belief in determinism, belief in dualism, self-control, cheating, utilitarian reasoning and the guilt experienced in response to the utilitarian dilemmas. MANCOVA, did not indicate significant main effect of the strength of the moral alarm, *F*(7,747) = 0.707, *p* = 0.666 or the level of explanation, *F*(7,747) = 0.721, *p* = 0.654 or an interaction effect *F*(7,747) = 1.015, *p* = 0.419. Also MANOVA indicated null results on the main effect of the strength of the moral alarm, *F*(7,752) = 358, *p* = 0.926, and the level of explanation, *F*(7,752) = 0.699, *p* = 0.673, and on the interaction effect, *F*(7,752) = 0.850, *p* = 0.546. The *post hoc* comparisons from MANOVA are reported in **Table 1**. The results remained null effects if the participants who reported disbelief in the manipulation were excluded from the analysis (*p* > 0.05). Complete descriptive statistics can be found in **Table 2**.

**Table 1:** *Post hoc*: The effects of the neuroscience manipulation, the moral alarm manipulation and their interaction (neuroscience ^∗^ moral alarm).

	Neuroscience			Moral alarm			Interaction		
Dependent variable	*df*	*F*	*p*	*df*	*F*	*p*	*df*	*F*	*p*
Self-control	1	0.08	0.779	1	0.42	0.516	1	<0.01	0.960
Free will	1	3.87	0.050	1	0.03	0.874	1	1.80	0.180
Determinism	1	0.15	0.696	1	0.66	0.418	1	0.03	0.864
Dualism	1	0.13	0.722	1	0.27	0.601	1	1.11	0.293
Guilt	1	0.02	0.903	1	0.08	0.783	1	1.36	0.243
Utilitarian reasoning	1	0.23	0.633	1	0.49	0.485	1	2.07	0.150
Cheating	1	0.11	0.745	1	0.40	0.528	1	<0.01	0.954


**Table 2:** Means, standard deviations, and bivariate correlations between all variables.

Variables	1	2	3	4	5	6	7	8
1. Gender	–							
2. Age	0.04	–						
3. Self-control	0.04	0.08^∗^	–					
4. Determinism	0.07	-0.05	-0.03	–				
5. Free will	0.02	0.04	-0.03	-0.06	–			
6. Cheating	0.04	0.06	-0.04	0.02	-0.07	–		
7. Guilt	-0.16^∗∗∗^	-0.02	0.05	-0.03	0.05	-0.08^∗^	–	
8. Utilitarian reasoning	0.17^∗∗∗^	-0.10^∗∗^	-0.04	0.00	-0.05	0.35	0.12^∗∗^	–
*M*		25.62	0.76	3.21	4.28	0.06	3.91	1.48
*SD*		7.51	0.43	1.26	1.28	0.25	1.25	0.92


*^∗^*p* < 0.05, ^∗∗^*p* < 0.01, ^∗∗∗^*p* < 0.005.*

To provide further evidence for the null results, the likelihood of the null model was compared to the alternative model by calculating the Bayes factors with BayesFactor package (Morey and Rouder, 2015) in R. In these analyses, the sample size was 762. Listwise deleting of the missing variables was used because currently, the BayesFactor package cannot handle any missing data. The Bayes factors are reported in **Table 3** against the alternative models that assume one of the main effects, their interaction, or both main effects and the interaction effect for each outcome variable. The default priors, which are described in detail in Rouder et al. (2012), were used. Shortly, these priors are multivariate generalizations of Cauchy priors for standardized effects; hence they are invariant in terms of the measurement unit. Bayes factors below 1 indicate that the null model is more likely than the alternative model, and thus all of the calculated Bayes factors support the null results. A Bayes factor of 0.1 means that the null model has become 10 times more likely than the alternative. The only exception to these low Bayes factors is the Level of Explanation’s effect on belief in free will, *B* = 0.54. While this Bayes factor means that the null model is two times more likely than the alternative, a Bayes factors of this size is sometimes considered weak evidence for the null model (e.g., Lakens et al., 2018). Thus the current data does not strongly discriminate between the null model and the alternative model regarding this effect; however, evidence for the lack of other effects is strong.

**Table 3:** Bayes factors for main effects, interaction, and main effects plus interaction.

Dependent variable	Neuroscience manipulation	Moral alarm manipulation	Neuroscience ^∗^ Moral alarm interaction	Moral alarm, Neuroscience manipulation + interaction
Self-control	0.08	0.10	0.01	<0.01
Free will	0.54	0.08	0.04	0.01
Determinism	0.09	0.11	0.01	<0.01
Dualism	0.09	0.09	0.01	<0.01
Guilt	0.08	0.08	0.01	<0.01
Utilitarian reasoning	0.09	0.10	0.01	<0.01
Cheating	0.08	0.10	0.01	<0.01


## Discussion

We predicted that lay people would be more likely to cheat and exhibit utilitarian reasoning after reading that their moral alarm was weak (H1), especially when this feedback was stated in neurobiological terms (H2). No effects, regardless of whether the feedback was neurobiological or cognitive, were observed. In particular, the participants who were attributed a weak moral alarm in the current study were no more likely to change their moral attitudes or behavior in response to the neurobiological explanation, suggesting that the communication of neuroscientific information does in fact not affect moral behavior. The feedback also exerted no effect on the expected mediators of the hypothesized effects; those mediators included self-control, belief in dualism, belief in free will, belief in determinism, and the guilt experienced in response to utilitarian dilemmas (H3). Therefore, the three hypotheses were unsupported.

This finding contrasts with evidence that challenges to free will promote immoral behavior (e.g., Baumeister et al., 2009). The discrepancy might be attributable to the fact that in the current study, neuroscience was communicated without reference to determinism. In this context, participants did not take the neuroscientific opportunity to excuse their weak moral alarm, consistent with previous null effects of challenging free will on cheating (Open Science Collaboration, 2015) and stealing (Monroe et al., 2017). This is an important finding because, in a separate study, we found that lay people may fear communicating neuroscience to offenders under certain circumstances, perhaps because of its anticipated implications for moral behavior (Blakey et al., unpublished). The current study suggests that this fear is empirically unjustified, since we did not find that it promoted immoral behavior.

These null effects are consistent with two studies directly asking offenders opinion on neuroscience: in interviews, serious young offenders tended to reject neuroscience and genetics, instead claiming the importance of social influences, and asserting their continued capacity for choice, responsibility and blame (Horstkötter et al., 2012, 2014). Horstkötter et al. (2014) also speculate two interesting incentives for offenders to retain attributions of choice: their dignity and their identity.

In respect to dignity, ‘even though [offending] may be a wrong choice, at least, it was their own choice’ (p. 8). In contrast, biology appears to offer an animalistic explanation that poses a greater threat to dignity. In respect to identity, proud offenders may seek to protect ‘their own, rebellious, voice,’ rather than perceive their behavior to be the predictable outcome of biological circumstances (Horstkötter et al., 2014, p. 8). One might expect this incentive to be particularly salient among psychopathic offenders, who construe moral responsibility as the opportunity to take credit, rather than receive blame, for their deviant behavior; in turn, their grandiose depiction of the self can be maintained (Hare, 1993). Contrary to neutralization theory (Sykes and Matza, 1957), therefore, people may wish to retain responsibility for deviant traits. The current participants may have sought to retain the ‘credit’ for their moral alarm, since even a weak moral alarm reaps certain advantages (Dutton, 2013). That credit might be retained by sustaining belief in free will and therefore responsibility for moral traits.

There are some methodological limitations that may have contributed to the observed null effects. First, 35% of the participants disbelieved or strongly disbelieved either one or both of the experimental manipulations. There was a particular reduction in the number of participants who believed the feedback of having a weak moral alarm for neurobiological reasons; perhaps, in pursuit of a positive view of the self, participants did not wish to believe that their own moral alarm was weak at the seemingly inherent level of biology. It is also possible that our results reflect the hypothetical nature of the moral choices that participants made, given evidence that hypothetical moral choices differ from real moral choices (FeldmanHall et al., 2012).

A further limitation of the present study regards the measure of dishonesty, known as the ‘die-under-the-cup’ task. The task has – in this case – resulted in a very low proportion of cheaters, perhaps suggesting that some participants realized the true purpose of the task and hence did not behave dishonestly. Importantly, however, the ‘die-under-cup’ task has previously been established as a valid measure of dishonesty (e.g., Halevy et al., 2013). Additionally, our particular test of self-control may have deviated from traditional tests by measuring a very particular form of self-control. In traditional self-control tasks, some reward is normally guaranteed and it is merely larger when people exert self-control. However, in the present study, participants had no such guarantee of reward; the reward was entirely dependent on a lottery. Hence, the task may have measured both the willingness to delay gratification and endure a high uncertainty of outcome across the same time frame. Future studies as such would benefit from having a true control group of participants who would, for example, read a passage of irrelevant text instead of performing the self-control task, in order to exclude any alternative explanations of the effects.

Furthermore, the sole difference between the two presented explanations of moral alarm was that one explanation referred to the ‘mind’ and the other to the ‘brain.’ Hence it is possible that the current participants did not make the distinction between ‘mind’ and ‘brain’ in the presented text, since no explanation contrasted ‘brain’ and ‘mind’ within one condition. However, in previous research, participants have shown implicit sensitivity to this subtle distinction (Bloom, 2004; Forstmann and Burgmer, 2015). It is also a novel contribution of the current research that the words ‘brain’ and ‘mind’ were presented, as opposed to neuroscientific jargon that refers to specific brain regions; the inclusion of jargon (only) in the ‘brain’ condition could have acted as a confounding variable.

In sum, while aspects of the study design could be improved, the hypotheses of this study were unsupported and Bayesian analyses confirmed the null results. This study therefore questions the notion that neuroscientific communications will cause people to behave immorally.

## Author Contributions

All authors listed have made a substantial, direct and intellectual contribution to the work, and approved it for publication.

## Conflict of Interest Statement

The authors declare that the research was conducted in the absence of any commercial or financial relationships that could be construed as a potential conflict of interest.

## Footnotes

1 applymagicsauce.com
